# “It’s a win for the clinic, it’s a win for the frontline, but, most importantly, it’s a win for the client”: Task Shifting HIV Prevention Services from Clinicians to Community Health Workers in Ontario, Canada

**DOI:** 10.1007/s13178-022-00721-y

**Published:** 2022-04-28

**Authors:** David J. Brennan, Maxime Charest, Aaron Turpin, Dane Griffiths, Barry D. Adam, John Maxwell, Keith McCrady, Robbie Ahmed

**Affiliations:** 1 Factor-Inwentash Faculty of Social Work, University of Toronto, 246 Bloor St. W, Toronto, ON M5S 1V4 Canada; 2 Faculty of Medicine, University of Ottawa, Ottawa, ON Canada; 3 Gay Men’s Sexual Health Alliance, Toronto, ON Canada; 4 Departments of Sociology, Anthropology and Criminology, University of Windsor, Windsor, ON Canada; 5 Ontario HIV Treatment Network, Toronto, ON Canada; 6 AIDS Committee of Toronto, Toronto, ON Canada; 7 2-Spirited Peoples of the 1st Nations, Toronto, ON Canada; 8 Alliance for South Asian AIDS Prevention, Toronto, ON Canada

**Keywords:** Task shifting, Men who have sex with men, HIV prevention, HIV testing, STBBI testing, PrEP, Sexual health

## Abstract

**Introduction:**

Despite strong evidence from low- and middle-income countries supporting the use of task shifting to provide quality, cost-effective HIV-related health services, this strategy has been adopted less widely in high-income countries such as Canada.

**Methods:**

In 2020, we conducted semi-structured interviews with 19 clinicians (e.g., psychologists, nurses, physicians) and 14 community health workers (CHWs) in Ontario to examine their perspectives on the prospect of shifting HIV/STBBI testing services and PrEP in Ontario, Canada. Interviews were transcribed and then analyzed using content analysis. A community consultation with key stakeholders was also performed to assess the validity of the findings.

**Results:**

There was substantial agreement between clinicians and CHWs with respect to shifting specific tasks related to HIV/STBBI testing and PrEP. In particular, most participants felt that rapid HIV testing could and should be provided by CHWs and that ASOs could be ideal sites for clients to obtain and use self-testing kits for STBBIs. Most respondents agreed that CHWs have the skills and expertise required to perform most non-clinical services related to PrEP (e.g., pre-counselling, follow-up, case management). The co-location of clinicians and CHWs could help support the development of task shifting initiatives.

**Conclusion:**

Findings indicate that there is enthusiasm among both clinicians and CHWs with respect to shifting HIV prevention services. Creative solutions are required to have a meaningful impact on HIV incidence in this population.

**Policy Implications:**

With adequate training and supervision, non-regulated CHWs should be allowed to provide certain HIV prevention services such as rapid HIV testing. A provincial, publicly funded program for PrEP is recommended.

## Introduction

Cisgender and transgender, 2-spirit, gay, bisexual, and other men who have sex with men (GBM) continue to be affected disproportionately by HIV in Canada. According to the most recent estimates, approximately 39.7% of new HIV infections across the country were among GBM, while an additional 3.4% were among GBM who inject drugs (Haddad et al., 2021). Several advancements have been made in recent years in the field of HIV prevention. For example, HIV pre-exposure prophylaxis (PrEP; the use of antiretroviral medication to prevent HIV infection) has been approved by Health Canada since 2016 and is highly effective at reducing risk of acquiring HIV (Grant et al., 2010; McCormack et al., 2016; Molina et al., 2015). Similarly, non-occupational HIV post-exposure prophylaxis (PEP) can be used following an exposure to HIV to help an individual prevent its acquisition (Tan et al., 2017). Individuals who are HIV-positive and have an undetectable viral load can also be confident that they cannot pass on the virus to others (Cohen et al., 2011; Rodger et al., 2019). Further, Canada has recently approved a self-testing kit for HIV (INSTI, 2021), which has been used to provide HIV testing services during the COVID-19 pandemic (O’Byrne et al., 2021a). Finally, some programs across the country, such as GetCheckedOnline by the British Columbia Centre for Disease Control, are also examining the feasibility of self-testing for other sexually transmitted and blood-borne infections (STBBIs) (GetCheckedOnline, 2016).

Although awareness of PrEP is high among Canadian GBM, its use remains low — particularly in urban settings (Brogan et al., 2019; Mosley et al., 2018; Rana et al., 2018). While generic versions of PrEP are available, part of the reason for this low uptake continues to be the high cost of the medication, especially in provinces where PrEP is not fully covered under provincial health insurance plans (Morgan et al., 2018). Some PrEP programs have been quite successful in dramatically reducing HIV incidence in large population centers, such as Vancouver (CBC News, 2019), London (PinkNews, 2017), and New South Wales (Collins, 2017). However, these programs were accompanied by large-scale public health campaigns, widespread accessibility, and financial coverage for the medication. There has also been a push in the last two decades to think about HIV prevention more holistically and to adopt combination HIV prevention approaches, which target not only biomedical interventions, but also tackle education and outreach, testing for HIV/STBBIs, as well as mental health and problematic drug use, among other determinants of health. Syndemics research (Ferlatte et al., 2018; Herrick et al., 2013, 2014; Stall et al., 2003) and minority stress theory (Frost et al., 2015; Meyer, 1995, 2003; Meyer & Frost, 2013) show that several systemic and individual psychosocial factors affect the likelihood of acquiring HIV. It follows that these factors need to be addressed in tandem to have a meaningful impact on reducing HIV rates. Given the limited accessibility of many of these services, creative solutions are needed to provide quality care to those who need it.

## What Is Task Shifting?

Task shifting is the rational redistribution of clinical tasks to trained health cadres with fewer credentials or qualifications (World Health Organization, 2008). As Callaghan et al. (2010) describe it, “The objective is a streamlined, rationalized chain of care that relieves pressure on each worker involved while maintaining quality standards for patients and increasing access to interventions.” In 2008, the World Health Organization (World Health Organization, 2008) published guidelines that endorsed task shifting as an appropriate and necessary way to address healthcare shortages and provide care in resource-limited or rural settings, particularly in low- to middle-income countries (LMICs). In other contexts, task shifting has also been utilized to provide health services for populations with more specific healthcare needs. Task shifting may involve the delegation of tasks from one healthcare provider to another (e.g., from physicians to nurses) or to non-clinical personnel, known alternatively as lay health workers, lay providers, peer support workers, paraprofessionals, frontline health workers, or community health workers (CHWs), among other terms (Barnett et al., 2018a, b; Raviola et al., 2019). This evidence-based approach to healthcare has been used in various settings and is conceptualized in a variety of ways to provide care where there may be no or limited services.

There is accumulating evidence, particularly from studies conducted in LMICs, that task shifting can be a cost-effective approach to increasing healthcare access and improving patient outcomes for a variety of health conditions (Barnett et al., 2018a, b; Callaghan et al., 2010; Fulton et al., 2011; Genberg et al., 2016; Grant et al., 2018; Hoeft et al., 2018; Kennedy et al., 2017; Mutch et al., 2017; Ryan et al., 2016; Seidman & Atun, 2017; Ti & Kerr, 2013; Zachariah et al., 2009). Callaghan et al. (2010) reviewed 84 studies looking at task shifting in sub-Saharan Africa. Their review concluded that there was some evidence that task shifting can be efficient and cost-effective, ultimately offering more accessible and affordable care to patients without sacrificing its quality. In fact, in some cases, task shifting resulted in better health outcomes among patient populations than the standard-of-care, including better retention in care, increased adherence to medication, and better viral suppression for individuals living with HIV (Callaghan et al., 2010). With respect to other HIV services, HIV-positive peers and CHWs have also been enlisted via task shifting opportunities with great success in providing point-of-care testing for CD4 + counts (Kaindjee-Tjituka et al., 2017), conducting interventions to increase patient linkages to care and adherence to antiretroviral therapies (Genberg et al., 2016), implementing increased access to HIV prevention services, and providing care for people who inject drugs (Ti & Kerr, 2013). However, despite this strong body of evidence accruing from LMICs, formal task shifting from clinicians to CHWs has rarely been employed as an approach to increase healthcare access and utilization in high-income countries such as Canada and requires further investigation.

## How Has Task Shifting Been Used in High-Income Countries?

Compared to LMICs, task shifting has been used much more sparingly in high-income countries. Nevertheless, available studies have shown it can be an effective way to provide increased access to services in resource-limited regions and increase patient engagement and retention in care (Hoeft et al., 2018). In terms of HIV/STBBI testing, there is some evidence that peer-based interventions and rapid testing in community spaces can increase the uptake of HIV testing (Kennedy et al., 2017), including among GBM (Lorenc et al., 2011; Shangani et al., 2017). For instance, community-based rapid HIV testing performed by CHWs has been used in France and Australia to increase access to testing for GBM (Leitinger et al., 2018; Lorente et al., 2013; Mutch et al., 2017; Ryan et al., 2017). In France, GBM who chose the community-based testing option had a similar risk profile to those who chose to attend the standard medical testing sites; however, they were more likely to have tested less frequently in the past 2 years (Lorente et al., 2013). In Australia, peer-led, community-based rapid HIV testing sites also attracted first-time testers and proved to be highly acceptable to those who used it (Leitinger et al., 2018; Mutch et al., 2017). However, the unavailability of STBBI testing alongside HIV testing was a barrier for some users, who preferred the convenience of testing for all STBBIs concurrently (Ryan et al., 2017). With respect to biomedical interventions, task shifting has been utilized in Canada to train nurses to prescribe PrEP and PEP under medical directives (i.e., documents signed by a physician to delegate a controlled act to trained personnel with fewer qualifications or credentials) (Charest et al., 2021; O’Byrne et al., 2018; O’Byrne et al., 2021a, b). However, aside from providing adherence support, counselling, or aiding in system navigation, task shifting of clinical services related to PrEP to CHWs has not been utilized in high-income countries (Vanhamel et al., 2020).

## Task Shifting Challenges

Some challenges associated with task shifting have been identified in the literature. For example, there is an upfront cost in terms of training new workers and ensuring they have access to the supports they need (Grant et al., 2018). A related challenge is providing adequate compensation for workers who take on new tasks, commensurate to the level of responsibility with which they are being charged (Zachariah et al., 2009). In addition, there is often rapid turnover of staff in community organizations such as AIDS service organizations (ASOs) and the rate of burnout is high in the field, which can make it difficult to retain personnel with specialized skills (Hoeft et al., 2018). Similarly, for task shifting to be effective, health cadres that take on new tasks need to have clearly delineated roles, as well as ongoing training and supervision (Callaghan et al., 2010; Fulton et al., 2011; Zachariah et al., 2009). There has also been some pushback from professional organizations and licensed clinicians in contexts where task shifting has occurred (Fulton et al., 2011; McPake & Mensah, 2008; Zachariah et al., 2009). In particular, healthcare professionals may be concerned that task shifting could result in compromising the quality of care that patients receive (Fulton et al., 2011; Zachariah et al., 2009), although several reviews have found that this is rarely the case when proper supports are in place (Barnett et al., 2018a, b; Fulton et al., 2011; Grant et al., 2018). Finally, governmental regulations can also make it difficult to shift certain tasks to non-clinical personnel. In Ontario, Canada, for example, prescribing medications, communicating a diagnosis, puncturing the skin, and psychotherapy are considered controlled acts which can be performed only by members of certain regulated professions such as physicians, psychologists, social workers, or nurses. Although some of these controlled acts can be delegated through a medical directive, related processes make it much more difficult to shift tasks such as HIV/STBBI testing or counselling to unlicensed CHWs.

## The Current Study

The current study was conducted to draw on the insights of healthcare providers and CHWs who serve GBM as to how task shifting could be utilized in Canada to improve access to combination HIV prevention services, specifically with respect to HIV/STBBI testing and PrEP. This study was part of a larger project entitled *Synergizing Health Interventions for Toronto GBM* (SHIFT) and utilized data from interviews to support and inform the implementation of task shifting interventions in Ontario, Canada. The current analysis focuses on clinician and CHW perspectives on which specific tasks related to HIV/STBBI testing and PrEP could be shifted from clinicians to CHWs working in ASOs, as well as anticipated benefits and challenges associated with task shifting these services. Specifically, we performed a thematic analysis on transcribed interviews to answer the following research questions: *Which HIV prevention tasks or services could be shifted to CHWs, particularly with respect to HIV/STBBI testing and PrEP? How might this task shifting occur, either in clinical or community spaces? What might be the benefits and challenges of task shifting these services?*

## Methods

### Methodology

Broadly, this research project employed a qualitative description approach for collecting, analyzing and interpreting the data (Bradshaw et al., 2017; Sandelowski, 2000; Sullivan-Bolyai et al., 2005). Qualitative description is used to provide rich descriptions of a phenomenon under study, typically using semi-structured interviews or focus groups with knowledgeable informants and by attempting to stay as close to participants’ own words as possible. The researchers then provide reasoned interpretations of the findings in simple language, which are supported by verbatim quotations. This approach has often been used in health research and can be particularly useful for practitioners and policy makers (Bradshaw et al., 2017; Sandelowski, 2000; Sullivan-Bolyai et al., 2005). According to Bradshaw et al. (2017), several criteria can be used to demonstrate rigor in the qualitative description approach. These include credibility (e.g., establishing rapport and a trusting relationship with participants), confirmability (e.g., describing participant demographics, engaging in member-checking), dependability (e.g., documenting the study procedures and processes), and transferability (e.g., purposeful sampling, rich description). Qualitative description does not preclude the use of other qualitative methods in the analysis of the data, which can be used to increase the rigor of analysis and interpretation. Qualitative description appeared to be a useful approach to the research study given the topic and the researchers’ close ties to clinicians and community.

### Procedure

This project was approved by the Research Ethics Board of the University of Toronto and University of Windsor. The study is a community-based implementation project consisting of four phases, the first two of which involved conducting interviews with clinicians and CHWs who work with GBM. The goal of these two first phases was to determine which aspects of combination HIV prevention services, specifically HIV/STBBI testing, PrEP, and mental health, could be shifted from licensed healthcare providers to CHWs. The third phase involved a community consultation process to ensure that findings concorded with the experiences and needs of research participants and key stakeholders who work in the field. In the fourth and current phase, we will be implementing a task shifting intervention based on our results. Although one of the foci of this project was on task shifting professional psychological services, the conversations about mental health were much more nuanced and complex than those about HIV/STBBI testing and PrEP. Given the additional complexities associated with providing psychotherapeutic services (e.g., the lack of universal coverage, the regulation of psychotherapy, the limited availability of services), as well as the subtleties associated with participants’ answers, findings from this portion of the interviews will be reported separately.

### Sample and Recruitment

In 2020, a convenience sample was recruited by contacting local clinicians and CHWs who work with GBM in southern Ontario, mainly in the Greater Toronto Area and other regions that already engaged in task shifting activities such as Ottawa. Most of the clinicians came from hospitals, clinics, public health, and private individual or group practices and the CHWs worked at ASOs in or around the Greater Toronto Area. Respondents were asked to identify other healthcare providers or CHWs that would be suitable for the study, who were also contacted by members of the research team. Eligibility criteria were the following: (1) being a clinician or CHW who was currently providing HIV prevention or sexual health services to GBM, (2) working in southern Ontario, (3) able to speak English, (4) being at least 18 years of age or older, and (5) being able and willing to provide informed consent. All participants provided written and verbal informed consent prior to participating in the research.

The final sample consisted of 19 clinicians (e.g., physicians, HIV specialists, nurses, psychologists, psychotherapists, clinical social workers, and pharmacists) and 14 CHWs who work with GBM. The mean age of the sample was 39.8 years (SD = 10.0). Most (*n* = 26) participants identified as men, but our sample also included women and genderqueer individuals. Some participants identified as transgender. Most (*n* = 16) participants identified as White, 5 as Asian (including East Asian, Southeast Asian, and South Asian), and the remainder identified with a variety of other racial/ethnic groups, including Black/African, Latin American, Middle Eastern, Indo-Caribbean, Jewish, Latin-European, First Nations, and Mixed Heritage. On average, the clinicians had spent 11.9 years (SD = 8.3) in their current role, 10.5 years (SD = 6.5) working with GBM, and 62.6% (SD = 24.9) of their clientele was made up of GBM. Comparatively, on average, the CHWs had spent 4.4 years (SD = 3.6) in their current role, 10.6 years (SD = 8.4) working with GBM, and 87.9% (SD = 16.3) of their clientele consisted of GBM. The tasks most commonly performed by CHWs can be found in Table 1.

**Table 1:** Tasks currently performed by community workers in ASOs

**Tasks**	**# of CHWs** **(*n*** **= 14)**
Safer sex education	14
Giving workshops	13
Outreach in person	12
Healthcare system navigation	11
Assessments/referrals for mental health	10
Assessments/referrals for social services	9
Assessments/referrals for physical health	8
Outreach online	8
Peer counselling	8
Sensitivity training	7
Case management	4
Point-of-care testing	3
Other	2

### Data Collection

Data were collected by conducting one-on-one, semi-structured interviews with healthcare providers and CHWs who work with GBM. Interviews were conducted in person, over the phone, or by Zoom. The study team developed two complementary interview guides for clinicians and CHWs. The interviews lasted 30 to 90 min, with most lasting ~ 1 h. The questions focused on participants’ experiences collaborating with other clinicians/CHWs; their experiences providing referrals to one another; which clinical tasks with respect to HIV/STBBI testing, PrEP, and mental health could be performed by CHWs; how CHWs could make clinical services more welcoming and accessible to a wider range of the GBM population; and how CHW skills and expertise could be leveraged to improve the delivery and accessibility of HIV prevention services. Some of the questions included the following: *How would you feel about CHWs performing pre- or post-test counselling for HIV/STBBIs? How could CHWs support the roll-out of PrEP? How can CHWs support clinicians who provide mental health services to GBM? Are there any skills or expertise that CHWs have that could benefit your practice? What kind of training or supervision would you (CHW) need to perform these types of tasks?* Data collection ended when the researchers felt that theoretical saturation had been reached.

### Data Analysis

Data were analyzed using NVivo 12. The analysis followed a qualitative approach (Creswell, 2009; Miles & Huberman, 1994) using thematic analysis (Glaser & Strauss, 1967) to answer the following research questions: *Which HIV prevention tasks or services could be shifted to CHWs, particularly with respect to HIV/STBBI testing and PrEP? How might this task shifting occur, either in clinical or community spaces? What might be the benefits and challenges of task shifting these services?* The researchers used content analysis (Hsieh & Shannon, 2005) to identify general themes that were relevant to the research questions at hand. Two research assistants (MC and AT) performed an initial coding of the first 10 interviews to determine whether the data were suitable for the analysis and to inform data collection in subsequent interviews. The research assistants compared codes and any discrepancies were resolved through discussion with the PI (DJB) until a consensus was reached. Using “constant comparison” (Glaser & Strauss, 1967; Goetz & LeCompte, 1984), the two research assistants then coded the remainder of the interviews and any differences in coding were once again resolved through discussion with the PI. This process was repeated until all inconsistencies were resolved and the data were organized into a narrative whole. This robust qualitative methodology was used to maximize the overall strength and trustworthiness of findings (Grinnel & Unrau, 2005). After data were analyzed, preliminary results were presented to the research team and to a group of study participants, CHWs, clinicians, and other key stakeholders as a form of member checking. This served as additional assurance that the findings were consistent with clinician and CHW experiences and were useful for those who might use them in the community.

## Results

There was substantial agreement between clinicians and CHWs in terms of the types of services that could be shifted to non-clinical personnel. Table 2 provides a summary of tasks which clinicians and CHWs agreed could be shifted to CHWs, as well as some of the associated benefits and challenges as expressed by participants. Generally, most respondents felt that simpler tasks which required less training, supervision, and/or clinical judgment could be suitable for CHWs to take on. This included, for example, rapid HIV testing and psycho-educational counselling for PrEP. Clinicians were more divided with respect to delegating controlled acts to unlicensed and non-clinical personnel. In general, most respondents were enthusiastic about the prospect of task shifting some HIV prevention services to CHWs. One physician expressed this succinctly: “I think it’s a win, win, win. It’s a win for the clinic, it’s a win for the frontline, but, most importantly, it’s a win for the client” (Physician 17). However, there was less agreement with respect to how this approach could be implemented most efficiently, especially with regard to services like PrEP, where a clinician necessarily needed to be involved.

**Table 2:** Tasks related to HIV/STBBI testing and PrEP that could be shifted from clinicians to CHWs

**Task**	**Benefits**	**Challenges**
**HIV/STBBI testing**		
Rapid HIV testing	• Greater, immediate access • Ability to reach underserved populations	• Ideally, should be paired with STBBI testing • Clients who test positive may need psychological support
Providing STTBI self-testing kits	• Greater, immediate access • Opportunity to educate clients and allay anxieties • Opportunity to talk to clients about harm reduction from a sex-positive lens	• Acquiring and funding self-testing kits • Storing samples and transporting them to laboratories
**PrEP**		
Pre-counselling and follow-up	• CHWs better positioned to discuss sexual risk and harm reduction • Clients may be more comfortable speaking openly with CHWs	• Requires a prescribing clinician • Might require co-location of clinician and CHW
Case management	• Ability to speak with clients about health system navigation, financing • Opportunity to engage in broader conversations about the role of PrEP in clients’ lives	• Time constraints on CHWs’ burdened schedules

### HIV/STBBI Testing

Nearly all respondents agreed that rapid HIV testing could be performed by CHWs in ASOs. In fact, one participant who had immigrated recently from a high-income country was surprised that rapid HIV testing was only performed by medical personnel in Canada, as CHWs in his country of origin performed this task. Some of the CHWs who were interviewed had also performed rapid HIV testing for clients or had been trained to do so under medical directives in the past. One of them spoke to the strong level of trust and respect that developed gradually with the clinicians with whom she was working at various clinics, once she had been in her position for some time. Participants noted a variety of potential benefits to CHWs performing rapid HIV testing, including reaching community members that may not be reached by mainstream sexual health services for GBM (especially trans, rural, and racialized GBM), reducing barriers to HIV testing, and being able to provide the service as soon as the need is expressed. One CHW mentioned that this could be particularly useful “for rural areas that are not as well served, I think it would be revolutionary” (CHW 7). Several CHWs mentioned that they would be happy to incorporate rapid HIV testing into their workload and that it would provide them with a tangible skill, which could in turn serve to increase their job satisfaction. One CHW, for example, expressed enthusiasm at the prospect of obtaining training to provide rapid HIV testing: “I’m interested in doing point of care [HIV testing]. It’s something that would not be a chore to me; it wouldn’t be like, oh my god there’s this extra thing I have to do. It’s like, no, I would be so stoked to do this because this is my area of interest” (CHW 3). A few participants mentioned, however, that most of their clients typically ask for HIV and STBBI testing at the same time. As such, offering only rapid HIV testing may be a deterrent for some of them. One participant expressed this concern: “I just find that only testing for HIV and not having the capacity to test for other STIs in the same go for me is a big limitation” (Social Worker 15). A public health nurse also voiced her fear that funding and services could be diverted from sexual health clinics should CHWs take on the bulk of rapid HIV testing services.

In addition, some of the clinicians, particularly physicians and psychologists, had concerns about CHWs performing post-test counselling or conferring a provisional diagnosis of HIV. Given the seriousness of HIV diagnoses, and the various reactions that clients may have in response to a positive test, participants felt that this might be too heavy an emotional burden for CHWs to bear. For example, one participant mentioned: “People have all sorts of reactions when they find out they are positive, so I think it would have to be like a trained mental health worker or clinician to give that” (Psychologist 16). Some participants also felt that a licensed mental health professional should be available should clients go into crisis. One physician, who admitted being more conservative in his views on medical directives, mentioned that he would not feel comfortable delegating any tasks to CHWs, including rapid HIV testing, unless there was a direct and well-established supervisory relationship between them: “Delegated medical services are intended when there’s a direct employee-employer relationship and that person is working directly under your supervision. There’s no way I would delegate, I mean, it’s like giving someone my medical license*.*” (Physician 1). Conversely, one physician felt that CHWs could be trained to perform HIV/STBBI testing, draw blood, interpret test results, confer a diagnosis, and even provide treatment and vaccinations for their clients: “I think frontline workers, if trained appropriately and given the support that they require and given the opportunity to reach out intermittently if needed, that they wouldn’t get themselves into big trouble” (Physician 17). This physician also mentioned that CHWs often perform these tasks in LMICs, which suggests that they could be trained to perform them in Canada. Several CHWs also mentioned that they had worked with clients who had recently been diagnosed with HIV or were in the room when their clients received a diagnosis of HIV, and therefore had provided post-test counselling in the past. In fact, many of them felt that they were quite well-equipped to provide support, pre- and post- test counselling, and referrals to services that their clients might need. One participant mentioned, “I think if you’re in this work, you probably, either through personal experience [or] you’ve been through enough tests that you feel like, I’ve kind of got this shtick down” (CHW 9).

There was less agreement, however, when it came to CHWs performing phlebotomy (i.e., drawing blood). Although some CHWs stated they would feel comfortable being trained to draw blood in order to perform STBBI screening for syphilis or hepatitis, many of them felt that this type of work should be left to healthcare providers. This was particularly the case when it came to swabbing genital or anal regions to detect chlamydia or gonorrhea. While there seemed to be less concern in terms of conferring a diagnosis of STBBIs (compared to HIV), most CHWs participants felt they were not comfortable drawing blood and especially performing genital swabs. Given that CHWs are often members of the communities they serve, they mentioned the dual relationships that already exist in their work and the need to maintain some professional boundaries. Some of the nurses we interviewed also felt that they could provide more in-depth information regarding HIV and STBBIs (compared to CHWs) given their educational background, especially if something unexpected were to come up during the testing process. As such, they stated that they would prefer that they or other licensed providers perform STBBI testing.

Nevertheless, should HIV/STBBI self-testing or home testing become more widely available in Canada, nearly all respondents stated that they would feel comfortable with CHWs handing out self-swabbing kits to their clients, explaining the procedures to them, and providing clients with a space within their organization where they could perform the swabs themselves. Although our interviews were conducted prior to the approval of self-testing kits for HIV by Health Canada in November of 2020 (INSTI, 2021), nearly all participants expressed that CHWs would be a vital resource for clients who choose to use this testing option. As one participant put it, “I don’t think it’s an option to not get trained on this, because whether we like it or not, people are going to increasingly be showing up after being tested alone because they’re going to Google HIV and be coming in to our services” (CHW 7). In particular, respondents felt that CHWs would be well-positioned to educate clients about the procedure, as well as allay any fears and anxieties that clients may have around testing. Participants also mentioned that ASOs could be ideal locations for clients to perform self-testing and access immediate resources should they receive a positive result, as well as obtain a referral to an appropriate healthcare provider. As one participant mentioned: “I think there’s also an opportunity to broker larger conversations about what contributes to anxiety and HIV stigma and how do we …? What kinds of behaviours have you engaged in and are there things you would do differently in the future around risk reduction and things like that?” (CHW 9) Many of the participants mentioned that CHWs might have more time than clinicians to engage in these broader discussions about sexual risk using a sex-positive and culturally competent approach. Some respondents indicated logistical challenges in terms of how these kits would be acquired and financed, how and where the samples could be stored, and how these would be transported to a laboratory for analysis.

### PrEP

All participants agreed that CHWs had a large role to play in terms of performing PrEP outreach and education with their clients, and most of the CHWs that we interviewed were already fulfilling that role. CHWs who worked within ASOs that served specific ethno-racial populations and/or newcomers to Canada indicated that education around PrEP was vital given misconceptions that were prevalent within their communities. However, in addition to educating community members, some participants felt that CHWs might be well-equipped to dispel myths around PrEP with family physicians and other clinicians as well. One participant talked about his negative experiences with some providers: “I’ve had some encounters with physicians, family doctors, whose personal opinion about PrEP is that it’s like devil worship. So, maybe some frontline workers could build capacity from physicians in explaining to them, okay people, there is a demand for PrEP, don’t be naïve” (Psychologist 11). Although family physicians can prescribe PrEP, several CHWs mentioned that some of their clients would not be comfortable accessing the medication through them, either because they were not out to them or did not feel comfortable discussing sexuality with their primary care providers. In addition to finding a suitable prescriber, the biggest challenge that nearly every participant identified with respect to PrEP delivery was the cost of the medication.

Many participants also mentioned that CHWs could be well-positioned to perform case management for PrEP. This might involve discussing with their clients the role that PrEP might play in their sex lives, navigating the healthcare system to access the medication, finding programs to finance it, and performing follow-up with clients. However, most of the CHWs that we interviewed stated that case management could not fit within their already overburdened workload and that they would need dedicated time within their schedule to be able to follow clients closely. One participant, for example, described his experience after he began engaging in PrEP system navigation with his clients: “So, I had very big clinics […] calling me and basically being, we need you to do this. And me, basically replying to them that this is just something that I do occasionally. I do it maybe max, four or five people a week. I cannot take on a caseload of trying to do PrEP access for an entire clinic, much less several clinics” (CHW 3). Although most of the CHWs discussed PrEP with clients through their work, and some had dedicated time for online outreach, many felt that case management would go beyond the scope of their role and take up too much of their time.

Aside from outreach, education, case management, and system navigation, many respondents thought that CHWs could safely and effectively perform most clinical tasks related to PrEP, with the exception of prescribing the medication. This included pre- and post-PrEP counselling, as well as dispensing scripts prescribed by a clinician for patients who have been on the medication for some time and have no complications. However, one of the main barriers to task shifting PrEP services was the need for a clinician to be involved at specific points in the process. Given that PrEP necessarily involves routine, quarterly testing for STBBIs and kidney function, a negative HIV test and a prescription, it would be nearly impossible for clients to meet solely with a CHW to obtain their medication. While many agreed that prescribing PrEP could easily be manualized and one physician even thought that CHWs could be trained to administer HIV/STBBI testing and interpret test results, several participants believed that the logistics of involving CHWs in prescribing PrEP might not be the most efficient use of resources. One psychologist commented: “I find that there’s nothing that puts an obstacle in more quickly than saying, now you have to go talk to somebody else” (Psychologist 8). Several CHWs mentioned that they had referred clients to PrEP services but most of them found it difficult to perform consistent follow-up with these clients. One solution that was offered repeatedly would be to have a CHW and a prescribing clinician working together in the same space. The CHW could then perform most of the pre-counselling, including discussing the benefits and drawbacks of the medication, finding financing programs for those who need them, and offer various strategies on how to use PrEP, such as daily or intermittent dosing. Some felt that CHWs might actually be better positioned than clinicians to have more open and comprehensive conversations about sexual risk and harm reduction approaches, given the limited time that physicians have and the liability that they must assume when counselling patients. Therefore, most respondents agreed that if a CHW and prescribing clinician were to be co-located, or at least working closely together, the healthcare provider might only be needed for medical tasks specifically (e.g., testing, prescribing) and could see patients quickly and efficiently.

## Discussion

Our findings suggest that there is strong enthusiasm for task shifting HIV prevention services in Ontario, Canada, especially with respect to rapid HIV testing and some clinical aspects of PrEP. Overall, there was much more agreement than disagreement between healthcare providers and CHWs in terms of the specific tasks that could be shifted to CHWs and the benefits that this could provide. Although participants described some potential challenges, they also identified a variety of advantages to task shifting, including reaching GBM with a broader range of sex/gender and ethno-racial identities, increasing capacity and satisfaction among CHWs, streamlining services, and providing more accessible options for GBM to engage with sexual health services.

Nearly all CHWs and healthcare providers voiced their support for CHWs to perform rapid HIV testing at ASOs. In fact, some of the CHW participants that we interviewed had been trained to deliver point of care HIV testing themselves. Given the success of peer-led and CHW-led models of rapid HIV testing in other high-income countries (Leitinger et al., 2018; Lorente et al., 2013; Mutch et al., 2017; Ryan et al., 2017), policy makers in Canada should look at the feasibility of amending regulations to allow CHWs to be trained and supported in providing this service for their clients. Supervision and training with a mental health professional could be useful should CHW clients go into crisis following a positive diagnosis. One of the limitations of providing rapid HIV testing in community settings would be the unavailability of concurrent STBBI testing services. However, there is already a successful example of a self-testing program for STBBIs in Canada (GetCheckedOnline, 2016) and research has shown that self-swabbing is comparable to clinician collected samples to detect oral, genital, and anal STBBIs (Yared et al., 2018). Correspondingly, several interviewees mentioned that ASOs could be ideal sites to obtain and perform self-testing for STBBIs. This would allow for a CHW to be present before and after the tests to counsel clients, explain the procedure, allay any fears or anxieties around testing, and provide referrals and resources. Since these interviews were conducted, a self-testing kit for HIV has been approved by Health Canada (INSTI, 2021) and its use is currently being investigated across the province of Ontario through the GetaKit program (O’Byrne et al., 2021b). Once again, ASOs could be ideal sites for clients to perform self-testing for HIV. Training should be provided to CHWs who are likely to encounter clients who have or are planning to perform self-testing for HIV.

Participants identified more challenges with respect to task shifting PrEP services. Although CHWs have been utilized in high-income countries to provide PrEP counselling, adherence support, and system navigation (Vanhamel et al., 2020), the fact that a clinician is required to interpret laboratory tests and prescribe the medication introduces some limitations on the amount of task shifting that can occur. In addition to outreach, education, and system navigation — which most CHWs in our study did already — participants mentioned that CHWs were well-suited to perform pre- and post-counselling for PrEP, follow-up with clients, provide case management, and have longer and broader conversations with clients about the role of PrEP in their lives. In fact, given that most CHWs are trained to provide services from a harm reduction lens, many participants felt that they might be better suited than physicians to have conversations about managing sexual risk and providing different options for clients to take PrEP (e.g., daily versus intermittent PrEP, PEP-in-pocket). One of the biggest challenges to CHWs performing case management was their inability to adopt additional responsibilities due to overburdened schedules. However, participants mentioned that co-locating CHWs with prescribers could result in a very efficient and client-centered approach to providing these services. In addition, some participants mentioned the stigma that clients had experienced regarding PrEP both in their communities and from healthcare providers themselves. CHWs could be well-suited to provide training and dispel myths about PrEP among community members and clinicians alike.

It is worth noting the resistance that may come from some licensed healthcare providers and professional organizations with respect to task shifting HIV prevention services from clinicians to CHWs. In our sample, one physician expressed trepidation towards delegating any clinical tasks to non-clinical personnel and one public health nurse voiced her concerns with respect to diluting the limited funding pool for and availability of sexual health services. To the former, there has previously been some pushback from professional organizations and healthcare providers in settings where task shifting has occurred (Fulton et al., 2011; McPake & Mensah, 2008; Zachariah et al., 2009). It may take some time for clinicians and CHWs to develop the mutual trust and respect necessary for CHWs to take on more clinical tasks and roles, as was described by one of our CHW participants who had been trained to provide rapid HIV testing at various clinics. To allay some of these anxieties, perhaps task shifting could first be implemented in settings where there can be a close supervisory relationship between healthcare providers and CHWs, so that clinicians feel assured that the quality of services delivered is maintained. In terms of diverting resources away from existing clinical services, some might argue that CHWs taking on tasks such as rapid HIV testing could reach different populations than are currently served by standard sexual health services. Given that more than 10% of people living with HIV in Canada remain undiagnosed (Public Health Agency of Canada, 2020), it is clear that current services are not reaching all those that they are meant to serve. Ultimately, the goal of task shifting is not to take away clinical tasks from licensed providers but rather to provide more accessible, culturally sensitive options for GBM to receive HIV prevention services. Task shifting could offer an entry point into the healthcare system, particularly for those who are currently underserved by mainstream services. Ideally, task shifting might allow clinicians and CHWs to work more closely together, develop a more streamlined referral network, and synergize their efforts towards eradicating HIV in Canada.

Our results indicate that creative solutions to providing sexual health services are required and desired by most clinicians and CHWs if we want to have a meaningful impact on HIV rates among GBM in Canada. However, there are larger structural issues that could curtail the widespread adoption of task shifting as a means to provide these services. For example, given limited funding for ASOs and high rates of turnover in the field, many CHWs may not be able to take on additional tasks. Dedicated positions in health clinics could partially address this problem, but these workers would need to be remunerated adequately based on the skills, training, and expertise required. The creation of gay men’s health hubs, such as HIM in Vancouver, the Fenway Institute in Boston, 56 Dean Street in London, and HQ in Toronto (which is set to be open this year), could be ideal sites for task shifting to occur. In addition, government funding dedicated to developing task shifting initiatives could help alleviate some of the upfront financial costs associated with training and supervising CHWs in performing these new tasks. Further, despite positive evidence regarding the successful delegation of prescribing PrEP and PEP from physicians to nurses (Charest et al., 2021; O’Byrne et al., 2018; O’Byrne et al., 2021a, b), no studies to date have investigated the possibility of delegating clinical tasks such as prescribing medication and interpreting laboratory test results to non-clinically trained health workers in high-income countries. Although physicians can delegate specific tasks to someone with whom they share a close working relationship, a strong foundation of trust and a high level of training and supervision would be required before attempting to delegate the act of prescribing PrEP to CHWs with no formal clinical training.

Finally, the cost of PrEP continues to be an obstacle to its use in Ontario (Morgan et al., 2018), with nearly all participants in our sample mentioning this as the primary barrier hindering their clients from accessing this intervention. Currently, despite generic versions of PrEP being available, not all insurance providers cover the cost of the medication fully in Ontario and it is not included under the provincial drug formulary, making it financially inaccessible for many. As mentioned above, PrEP programs which have made a significant impact on HIV incidence rates among GBM in Canada and internationally, such as those in Vancouver (CBC News, 2019) London (PinkNews, 2017), and New South Wales (Collins, 2017) all offered free access to the medication, which likely contributed to their success. The Government of Ontario should consider creating a publicly funded PrEP program so as to help increase its use among our most marginalized communities and ease the burden of HIV in this population.

## Strengths and Limitations

To our knowledge, this is one of the first studies to examine clinician and CHW perspectives on the prospect of task shifting HIV prevention services in a high-income country. The inclusion of both healthcare providers and CHWs allowed us to compare their responses, providing us with a more fulsome understanding of which HIV prevention tasks could be shifted and how task shifting could occur in this context. The sample was diverse in terms of sex/gender, ethno-racial identity, and healthcare professions, and CHW participants came from organizations that cater to a variety of ethno-racial GBM groups, improving the generalizability of our results. We also performed a community consultation with study participants, community members, and key stakeholders, lending more credibility to the ecological validity and utility of our findings. However, this study also has limitations. Given that the original focus of the study was on Toronto, Canada, few of our respondents came from smaller cities or rural regions. Their perspectives could have added some richness to our findings, especially given that healthcare services are typically more limited in remote regions. In addition, the use of a convenience sample could have biased some of our results.

## Conclusion

It is clear from our findings that there are many opportunities for healthcare providers and CHWs to synergize their efforts towards providing more accessible and culturally competent HIV prevention services for GBM. Researchers, community organizations, and policy makers should work together to harness the knowledge, skills, and expertise of CHWs to streamline sexual health services for this population.

## Data Availability

To protect the confidentiality of interview participants, interview data will not be published.

## Code Availability

N/A.
